# Off-grid field-deployable molecular diagnostic platform for malaria surveillance

**DOI:** 10.1186/s13071-025-06779-y

**Published:** 2025-04-23

**Authors:** Madhavinadha Prasad Kona, Armel N. Tedjou, Mary Kefi, Francesco Buongiorno, Charles S. Wondji, George Dimopoulos

**Affiliations:** 1 W. Harry Feinstone Department of Molecular Microbiology and Immunology, Bloomberg School of Public Health, Johns Hopkins University, Baltimore, MD 21205 USA; 2 Centre for Research in Infectious Diseases (CRID), P.O. Box 13591, Yaoundé, Cameroon; 3 Hyris Research Center, Viale Lancetti, 19, 20158 Milan, Italy; 4 Department of Vector Biology, Liverpool School of Tropical Medicine, Pembroke Place, Liverpool, L35QA UK

**Keywords:** Malaria, *Plasmodium falciparum*, *Anopheles gambiae*, TaqMan qPCR, Diagnostics, Cameroon

## Abstract

**Background:**

Malaria, a major global health concern, continues to cause substantial morbidity and mortality, particularly in tropical regions. Traditional malaria diagnostic methods such as microscopy and quantitative polymerase chain reaction (qPCR) are effective but face challenges in field settings because of their requirement for laboratories with specialized equipment and trained personnel. This study presents the development and validation of a portable, cost-effective, field-deployable real-time qPCR platform for detecting *Plasmodium* species.

**Methods:**

Field-compatible DNA isolation was performed using DNAzol, and TaqMan probes targeting 18S ribosomal RNA (rRNA) were employed to detect five *Plasmodium* species—*P. falciparum*, *P. vivax*, *P. malariae*, *P. ovale*, and *P. knowlesi*—using the bCUBE qPCR platform. In vitro-cultured *P. falciparum* and experimentally infected *Anopheles gambiae* were used to quantify *P. falciparum* infections, with infection prevalence compared to microscopy. The bCUBE qPCR system was also evaluated under field conditions to detect *P. falciparum* infections in field-collected *An. gambiae* mosquitoes.

**Results:**

The bCUBE qPCR demonstrated a strong linear correlation (*R*^2^ = 0.993) with a standard laboratory qPCR machine for detecting *P. falciparum* infections. It successfully detected as few as 0.5 parasites/µl of blood, one oocyst in mosquito guts, and 5–10 sporozoites in salivary glands. It was also capable of discriminating between *P. falciparum*, *P. vivax, P. malariae*, *P. ovale*, and *P. knowlesi.* Field evaluations in Cameroon confirmed its accuracy in identifying *P. falciparum* in mosquito samples, with same-day results. The capability of the bCUBE qPCR system to detect infections in both individual and pooled mosquito surveillance further highlights its potential for in-field large-scale malaria monitoring surveillance.

**Conclusions:**

The bCUBE qPCR system offers a portable, sensitive, and scalable solution for malaria diagnostics, enabling real-time surveillance in resource-limited settings. Its ability to provide rapid, on-site results reduces the need for centralized laboratory testing, facilitating timely decision-making in malaria control programs.

**Graphical Abstract:**

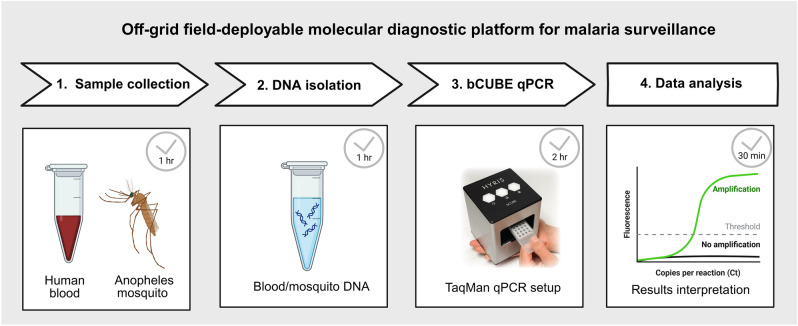

## Background

Malaria, one of the most serious infectious diseases globally, is associated with significant morbidity and mortality, especially in tropical and subtropical regions [1, 2]. Caused by *Plasmodium* parasites, it is transmitted primarily through the bite of an infected *Anopheles* mosquito. Despite substantial efforts to control and eliminate this disease, it continues to be a serious public health problem, with hundreds of millions of cases and over 600,000 deaths reported annually [3].

Accurate and rapid diagnostics are crucial for managing and controlling malaria. Traditional methods such as microscopy and rapid diagnostic tests (RDTs) have been a vital part of malaria control programs [4]. Microscopy, considered the gold standard for malaria diagnosis [5], is used to detect blood-stage parasites in humans via blood smears and to detect mosquito-stage parasites, i.e., oocysts, in dissected mosquito midguts and sporozoites in salivary glands [6]. However, this method requires highly trained personnel and laboratory facilities, making it impractical in many resource-limited settings. RDTs, which detect specific antigens from *Plasmodium* species in a patient’s blood, offer a quicker and simpler alternative. However, the sensitivity and specificity of RDTs can vary, depending on the parasite density and genetic variations between *Plasmodium* strains, and they may fail to detect low parasitemia or non-*falciparum* species, leading to false-negative results, in addition to false negatives due to histidine-rich protein (HRP)2/3 deletion [7, 8].

Molecular diagnostic techniques such as polymerase chain reaction (PCR) and real-time quantitative PCR (qPCR) offer a more sensitive approach that is capable of detecting low-level parasitemia and mixed-species infections that are often missed by microscopy or RDTs [9]. The *Plasmodium* 18S ribosomal RNA (rRNA) gene is a preferred target because it occurs in multiple copies per genome (5–7), enhancing the assay’s sensitivity [10]. Real-time qPCR methods such as TaqMan™, which utilize fluorescent probes to detect target sequences, are particularly effective for quantifying *Plasmodium* DNA with high precision [11, 12]. However, these methods often require sophisticated infrastructure and are not easily adapted to field conditions.

To address these limitations, several advanced point-of-care diagnostic tools have been developed, including loop-mediated isothermal amplification (LAMP) and biosensor-based techniques [13–15]. While these approaches are promising, they still face significant challenges that have limited their widespread adoption in low-resource settings. For example, LAMP can show cross-reactivity with other pathogens, and both LAMP and biosensor-based methods often require specialized training and equipment, making them difficult to implement in resource-constrained areas where access to advanced infrastructure and skilled personnel is limited [16, 17].

Given the challenges of traditional and molecular diagnostics, the need for a portable, field-deployable qPCR platform that can deliver accurate results rapidly in low-resource settings has become increasingly important. In this study, we developed and validated a novel portable real-time PCR platform, the bCUBE (Hyris), for detecting *Plasmodium* species. The platform was validated for detecting *P. falciparum* in laboratory-cultured asexual blood-stage parasites, experimentally infected mosquitoes, and field-collected mosquito vectors.

## Methods

### Preparation of infected blood samples from *P. falciparum* in vitro blood-stage cultures

In vitro *P. falciparum* (NF54 strain) asexual blood-stage cultures were obtained from the Johns Hopkins Malaria Institute Core Facility. Parasitemia was determined by examining Giemsa-stained blood smears under a light microscope [18]. The infected blood culture was then diluted with uninfected human whole blood to prepare a series of mock *P. falciparum* whole-blood mixtures with varying parasite concentrations of 50,000, 5000, 500, 50, 5, 0.5, and 0.05 parasites/µl. For each dilution, a total volume of 100 µl was prepared, and 10 µl of each sample was used for subsequent DNA extraction.

### Preparation of *P. falciparum*-infected mosquito samples

Mature *P. falciparum* NF54 gametocytes and *Anopheles gambiae* mosquitoes were obtained from the Johns Hopkins Malaria Institute Core Facility. Mature gametocyte cultures exhibiting high levels of exflagellation were used to infect *An. gambiae* mosquitoes via standard membrane feeding assays (SMFAs) as described previously [18–20]. In brief, gametocyte cultures were diluted with fresh red blood cells (RBCs) to achieve two different gametocytemia levels—0.3% (high) and 0.01% (low)—and an equal volume of pre-warmed heat-inactivated AB+ serum was added to both cultures. Then, 4- to 5-day-old *An. gambiae* mosquitoes, starved by feeding for 6–8 h on water-soaked cotton, were fed these cultures via glass membrane feeders. After blood-feeding, the unfed mosquitoes were removed, and the remaining fed mosquitoes were maintained at 25–26 °C.

Nine days after the blood meal, half of the mosquitoes were dissected, and midguts were examined by microscopy to determine the oocyst load. The remaining mosquitoes were kept until day 15, when their salivary glands were dissected to assess sporozoite loads by microscopy. From the same batch, 25 individual mosquitoes were taken for bCUBE qPCR analysis to detect *P. falciparum* infection intensities at both the oocyst and sporozoite stages. For oocyst detection, DNA was extracted from the mosquito abdomen, while for sporozoite detection, only the head and thorax were used. Samples were collected from three independent experiments, each performed in duplicate, to compare the results of the bCUBE qPCR with the gold-standard microscopy method.

### *Plasmodium falciparum* detection in pooled mosquito samples

Laboratory-reared *An. gambiae* mosquitoes, both infected with *P. falciparum* and uninfected, were used to create various mosquito pools. Five pooled sample sets were prepared with ratios of infected to uninfected mosquitoes of 1:5, 1:10, 1:15, 1:20, and 1:25. Each pool contained one *P. falciparum*-infected mosquito (15 days after infection) mixed with 4, 9, 14, 19, or 24 uninfected mosquitoes, respectively. DNA was extracted from these pools, and qPCR analysis was performed using the bCUBE platform. DNA was also extracted from a sample containing only 25 uninfected mosquitoes (as a negative control) and from a sample with a single *P. falciparum*-infected mosquito (as a positive control). The results were analyzed to determine the platform’s ability to detect low levels of *P. falciparum* infection in pooled mosquito samples.

### DNA isolation using DNAzol reagent

DNA was extracted from both *P. falciparum* blood-stage cultures and infected mosquito samples using DNAzol reagent (Invitrogen) according to the manufacturer’s protocol, with minor modifications to optimize field applicability. For blood samples, 10 µl of each *P. falciparum*-infected blood sample was transferred to an individual eight-strip PCR tube. To each tube, 100 µl of DNAzol reagent was added, and the mixture was gently pipetted up and down several times to lyse the cells. The samples were incubated at room temperature for 5 min. After cell lysis, 100 µl of absolute ethanol was added to precipitate the DNA. The mixture was gently inverted several times and incubated for 10 min at room temperature. The samples were then centrifuged in a tabletop PCR-strip mini centrifuge for 8 min. The resulting DNA pellet was washed twice with 0.2 ml of 75% ethanol, each wash step involving a 3-min centrifugation in the mini centrifuge. Between washes, the supernatant was discarded, and 0.2 ml of fresh 75% ethanol was added to the pellet. The pellets were air-dried and resuspended in 50 µl of 8 mM NaOH for subsequent analysis.

For mosquito samples, various mosquito tissues were processed, depending on the stage of infection. For oocyst detection, DNA was extracted from the mosquito abdomen, whereas for sporozoite detection, only the head and thorax were used. Individual mosquitoes or pooled mosquito samples were collected in 1.5-ml centrifuge tubes and homogenized with a pestle in DNAzol reagent. The volume of DNAzol used varied according to the pool size: 100 µl for pools of 1–5 mosquitoes, 250 µl for 6–10 mosquitoes, and 500 µl for 11–25 mosquitoes. The homogenates were then centrifuged using a tabletop microcentrifuge tube mini centrifuge for 2 min, and 100 µl of the supernatant was transferred to an eight-strip PCR tube for subsequent processing.

The next steps, including the addition of 100 µl of absolute ethanol to precipitate the DNA and the subsequent ethanol wash steps, were the same for all samples, irrespective of the initial volume of DNAzol used for homogenization. The DNA pellet was washed twice with 0.2 ml of 75% ethanol, air-dried, and finally resuspended in 50 µl of 8 mM NaOH. The use of eight-strip PCR tubes, instead of standard 1.5-ml centrifuge tubes, streamlined handling in field conditions by minimizing sample processing time and reducing the need for high-speed centrifugation.

### bCUBE-based TaqMan qPCR assays

A portable bCUBE 3.0 thermocycler (Hyris) was used in this study, along with 16- or 36-well cartridges (Hyris). The data analysis was performed using the Hyris data analysis platform. For *Plasmodium* species detection, previously published primers and probes targeting the 18S rRNA gene were used [21] and synthesized by Eurofins Genomics (USA).

TaqMan qPCR was carried out in a 20 µl reaction volume consisting of 0.4 µM *Plasmodium* species-specific forward and reverse primers, 0.2 µM FAM-labeled probe specific for *P. falciparum*, 1× probe master mix (Qiagen), and 5 µl of template DNA. The thermal cycling conditions were as follows: initial denaturation at 95 °C for 10 min, followed by 40 cycles of 95 °C for 15 s and 60 °C for 1 min. Each assay was performed in three independent biological replicates. Positive controls included *P. falciparum* DNA; uninfected human blood and mosquito DNA were used as negative controls. The sequences for the primers and probes used in this study are given in Table 1.

**Table 1:** Primers and probes used to detect *Plasmodium* species targeting 18S rRNA genes (Shokoples et al. [21])

*Plasmodium* spp.	Primer	Sequence (5′-3′)	Fluorescent label
Simplex PCR			
*P. falciparum*	Fal-F	CCG ACT AGG TGT TGG ATG AAA GTG TTAA	FAM-MGBEQ
	Fal-R	AAC CCA AAG ACT TTG ATT TCT CAT AA	
	Fal Probe	AGCAATCTAAAAGTCACCTCGAAAGATGACT	
Duplex PCR			
*P. falciparum*	Fal-F	CCG ACT AGG TGT TGG ATG AAA GTG TTAA	FAM-MGBEQ
	Fal-R	AAC CCA AAG ACT TTG ATT TCT CAT AA	
	Fal Probe	AGCAATCTAAAAGTCACCTCGAAAGATGACT	
*P. vivax*	Viv-F	CCG ACT AGG CTT TGG ATG AAA GAT TTTA	HEX-MGBEQ
	Viv-R	AAC CCA AAG ACT TTG ATT TCT CAT AA	
	Viv Probe	AGCAATCTAAGAATAAACTCCGAAGAGAAAATT	
Triplex PCR			
*P. malaria*	Mal-F	CCG ACT AGG TGT TGG ATG ATA GAG TAAA	FAM-MGBEQ
	Mal-R	AAC CCA AAG ACT TTG ATT TCT CAT AA	
	Mal Probe	CTATCTAAAAGAAACACTCAT	
*P. ovale*	Ova-F	CCG ACT AGG TTT TGG ATG AAA GAT TTTT	HEX-MGBEQ
	Ova-R	AAC CCA AAG ACT TTG ATT TCT CAT AA	
	Ova Probe	CGAAAGGAATTTTCTTATT	
*P. knowlesi*	Knw-F	CTA AAA TGC GCA CAA AGT CGAT	CY5-MGBEQ
	Knw-R	GCA GTT AAA ACG CTC GTA GTT GAA	
	Knw Probe	CGGAGGCATCAGTTAT	

### Laboratory-standard qPCR

A standard benchtop StepOnePlus Real-Time PCR System (Applied Biosystems, Thermo Fisher Scientific) was used for comparative analyses to validate the performance of the portable bCUBE qPCR system. The same reagents and reaction conditions were used as described for the bCUBE qPCR, except for a reaction volume of 15 µl and 2 µl of DNA template per reaction.

This standardized protocol ensured consistency between the two platforms, allowing for a direct comparison of the sensitivity and efficiency of *P. falciparum* detection between the portable bCUBE and the conventional StepOnePlus qPCR system.

### Preparation and detection of mixed *Plasmodium* species samples

To detect all five human *Plasmodium* species using a TaqMan probe-based multiplex bCUBE qPCR system, we obtained plasmids containing the 18S rRNA genes of *P. vivax*, *P. malariae*, and *P. ovale* from the Biodefense and Emerging Infections Research Resources Repository (BEI Resources; MRA-178, MRA-179, MRA-180). The 18S rRNA gene of *P. falciparum* was amplified from genomic DNA provided by the Johns Hopkins University (JHU) Malaria Parasite Core Facility, and the *P. knowlesi* 18S rRNA gene was amplified from genomic DNA obtained from BEI Resources (MRA-456G). These genes were cloned into pCR2.1-TOPO plasmids and PGMT vectors, respectively, according to the manufacturer’s instructions. The 18S rRNA gene of *P. falciparum* and *P. knowlesi* was amplified using genus-specific primers by nest1 PCR, following the protocol described by Snounou et al. and Mehlotra et al. [22, 23]. Positive clones for all five *Plasmodium* species were selected, and the corresponding plasmids were isolated to optimize the detection of mixed infections using the bCUBE qPCR.

Two multiplex PCRs were designed. The first was a duplex PCR to detect *P. falciparum* and *P. vivax* using FAM- and HEX-labeled fluorescent probes, respectively. The second was a triplex PCR to detect *P. malariae*, *P. ovale*, and *P. knowlesi* using FAM-, HEX-, and CY5-labeled fluorescent probes. To simulate mixed infections, plasmids containing each *Plasmodium* species’ 18S rRNA gene were mixed in various combinations, with one plasmid at a concentration of 10^3^ copies and the other plasmids at concentrations ranging from 10^3^ to 10^6^ copies. These pooled plasmids were used as templates for the bCUBE qPCR, and Ct values were determined based on duplicate samples.

The duplex bCUBE qPCR was performed with a reaction mixture containing 0.2 µM of *P. falciparum*-specific FAM-labeled probe and *P. vivax*-specific HEX-labeled probe, along with species-specific primers and 1× probe master mix. The triplex PCR included FAM, HEX, and CY5 probes for detecting *P. malariae*, *P. ovale*, and *P. knowlesi* in the same reaction. Both reactions were prepared with 0.4 µM species-specific forward and reverse primers, 0.2 µM fluorescent-labeled probes, 1× probe master mix, and 5 µl of template DNA in a 20 µl total reaction volume. PCR amplification was carried out with the following conditions: initial denaturation at 95 °C for 10 min, followed by 40 cycles of 95 °C for 15 s and 60 °C for 1 min.

### Field study

To assess the field applicability of the bCUBE qPCR diagnostic platform, we conducted a pilot study in Cameroon, Africa. The portable bCUBE instrument was transported directly to the field, and all procedures, including mosquito collection, morphological identification, DNA isolation, qPCR setup, and data analysis, were performed on-site. Mosquitoes were collected in Mangoum (5°29′09.2″N, 10°35′20.8″E), located in the western region of Cameroon at an altitude of 1054 m above sea level. Mangoum features both extensive manual and mechanized agricultural systems that produce spices, vegetables, and cereals. This region experiences four seasons: two rainy seasons, occurring from March to June and September to November, and two dry seasons, spanning from December to February and July to August.

The collected mosquitoes were identified morphologically as *An. gambiae* using the identification keys of Gillies and Coetzee [24]. In the field, the bCUBE instrument was set up to detect *P. falciparum* infections in the collected mosquitoes.

The mosquitoes were dissected to separate the blood-fed abdomen from the head + thorax, and each section was placed in an individual 1.5-ml tube for DNA isolation, using DNAzol reagent as described above and squash buffer as described previously [13] to compare the field-compatible isolation protocol. A total of 26 field-collected *An. gambiae* mosquitoes were tested for *P. falciparum* infection using the portable bCUBE qPCR. The mosquitoes were first dissected as described above to separate the head + thorax from the abdomen, and two different DNA isolation methods were used. DNA was extracted from 20 individual mosquito samples (40 separate abdomen and head + thorax samples) using the DNAzol reagent. DNA was extracted from the remaining six mosquitoes using the squash method. All DNA extractions and bCUBE qPCR assays were performed on-site within 4–5 h to simulate a field-based workflow.

The TaqMan probe master mix was prepared for each reaction by combining the probe master mix, species-specific forward and reverse primers, and a 5′ FAM-labeled probe targeting the *P. falciparum* 18S rRNA gene, according to the previously described simplex qPCR protocol. The bCUBE machines were connected via a mobile hotspot to initiate the run, and the test results were analyzed directly in the field. Following the bCUBE qPCR analysis, the extracted DNA was transported to a main laboratory in Yaoundé, Cameroon, where conventional benchtop qPCR (Agilent MX3000P) was performed using the same DNA samples in order to compare the sensitivity between the two platforms. The qPCR assays targeted the *P. falciparum* 18S rRNA gene using TaqMan probes and species-specific primers.

### Statistical analysis

The prevalence of *Plasmodium* infection was visualized using bar graphs generated with GraphPad Prism 10 software. Statistical differences between three independent biological replicates were assessed, and the data were combined for analysis. The significance of differences in infection prevalence (the proportion of infected mosquitoes out of the total examined) between the bCUBE qPCR and microscopy methods was determined using a non-parametric Mann–Whitney test. Data from three biological replicates are presented in all figures. Data points for the standard curve were plotted using a scatter plot, and the correlation coefficient (*R*^2^) was calculated to evaluate the linearity and fit of the standard curve.

## Results

### Validation of the portable bCUBE qPCR machine against a standard laboratory qPCR system

**Fig. 1:**
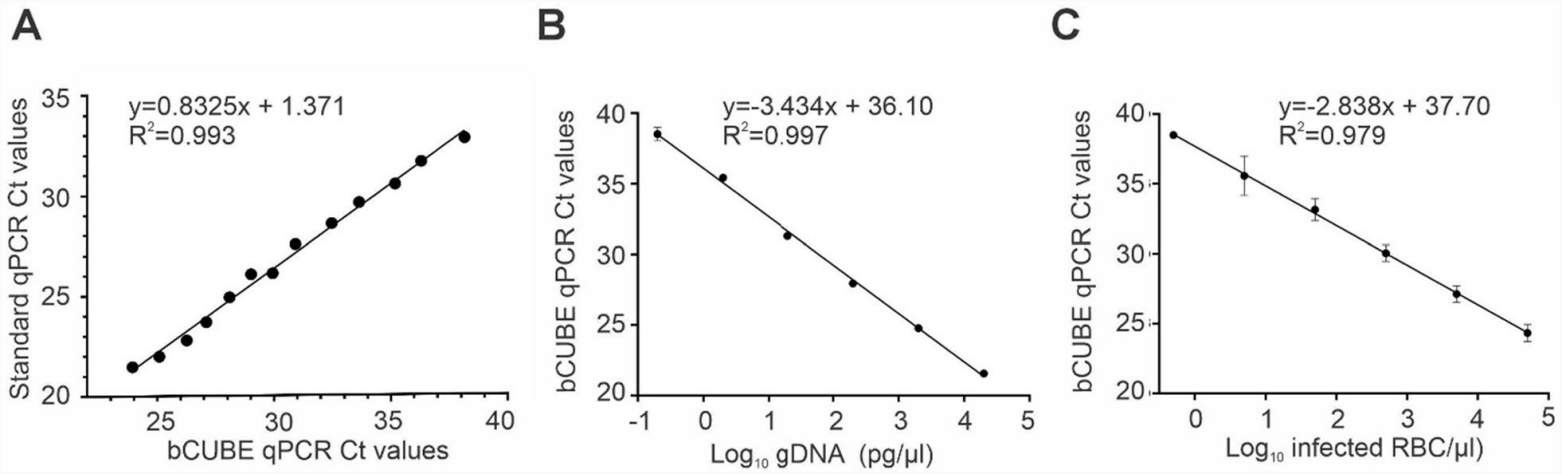
Comparative analysis of two qPCR machines and bCUBE-qPCR-based standard graphs for detecting *P. falciparum*. **A** Correlation curve depicting the comparison of *P. falciparum* 18S rRNA gene detection in serially diluted DNA using bCUBE and laboratory-standard real-time qPCR. The correlation coefficient was calculated for twofold serially diluted *P. falciparum* DNA. The bCUBE qPCR Ct values are plotted on the *x*-axis and standard qPCR Ct values on the *y*-axis. **B** Standard curve showing the correlation of bCUBE Ct values with 10-fold serially diluted *P. falciparum* genomic DNA (20 ng to 0.2 pg). **C** Standard curve showing the correlation of bCUBE Ct values with 10-fold serially diluted *P. falciparum-*infected blood (from 5 × 10^4^ to 0.5 parasitized RBCs/μl). The slope of each line represents [−1/log_10_ (PCR efficiency)] for a TaqMan probe assay. *R*^2^ represents the correlation coefficient of a slope. Ct, cycle threshold

To validate the performance of the portable bCUBE qPCR, a comparative analysis was conducted using the laboratory-standard StepOnePlus Real-Time qPCR System (Applied Biosystems, Thermo Fisher Scientific). Both systems were assessed using TaqMan qPCR assays targeting the *P. falciparum* 18S rRNA gene. Serial dilutions of *P. falciparum* genomic DNA were prepared and tested using the same qPCR master mix and primers on both platforms. The correlation analysis between the cycle threshold (Ct) values of the two machines showed a strong linear association, with an *R*^2^ value of 0.993, indicating a strong correlation between the bCUBE qPCR and the standard qPCR system results (Fig. 1A).

Next, we evaluated the detection limit of the bCUBE qPCR assay using 10-fold serial dilutions of *P. falciparum* DNA, from 20 ng/µl down to 0.2 pg/µl. The bCUBE qPCR demonstrated high sensitivity, accurately detecting *P. falciparum* DNA concentrations as low as 1 pg/µl, with a strong linear fit (*R*^2^ = 0.997) (Fig. 1B). We also assessed the detection limit by serially diluting *P. falciparum*-infected blood stage cultures in human whole blood to achieve parasite concentrations as low as 0.5 parasites/µl. The bCUBE qPCR assays successfully detected *P. falciparum* at concentrations as low as 0.5 parasites/µl, demonstrating high sensitivity with an *R*^2^ value of 0.979 (Fig. 1C).

These results confirm that the portable bCUBE qPCR assays perform comparably to a conventional laboratory-standard qPCR machine in detecting *P. falciparum* infections, even at low concentrations. They also strongly suggest that the bCUBE qPCR platform can serve as a reliable and sensitive tool for field-based malaria diagnostics, particularly in low-resource settings where early detection of low parasite densities is critical for effective disease management and control.

### Strong correlation between bCUBE-qPCR and microscopy in detecting infected mosquitoes

To evaluate the performance of the bCUBE qPCR system in detecting *P. falciparum* infections in mosquito vectors, we compared its results to those obtained using microscopy, the gold standard for detecting oocyst and sporozoite stages in infected mosquitoes. Three independent experiments were conducted using *An. gambiae* mosquitoes (*n* = 70–100), which were infected with *P. falciparum* by feeding on low- and high-gametocytemia cultures to establish various infection intensities in mosquitoes. Each cohort was divided into two groups: The mosquitoes in one group were analyzed individually using the bCUBE qPCR system, while those in the other group were evaluated by microscopy to determine the infection prevalence in midgut (oocyst) and salivary gland (sporozoite) samples at 9 and 15 days post-infection.

**Fig. 2:**
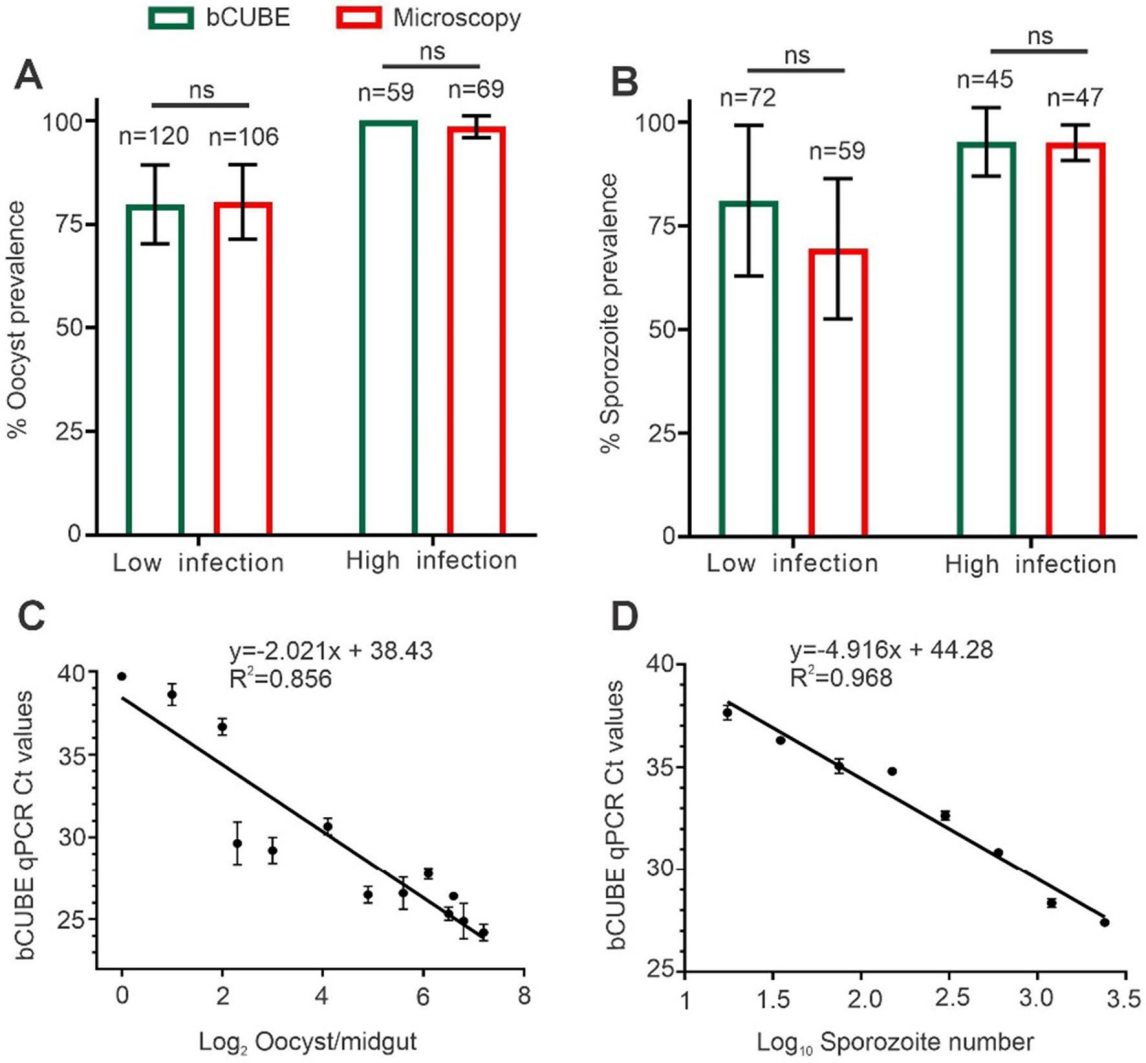
*Plasmodium falciparum* infection prevalence in *An. gambiae* as detected by bCUBE qPCR versus microscopy. Bar graph depicting the *P. falciparum* infection prevalence (± SD) of oocysts (**A**) and sporozoites (**B**) at 9 and 15 days post-infectious blood meal in *An. gambiae,* respectively. Three independent experiments were conducted using *An. gambiae* mosquitoes infected with *P. falciparum* at low and high gametocytemia to establish various infection intensities. Statistical analyses were performed using the Mann–Whitney test. A standard correlation graph showing the detection of oocyst load in microscopy confirmed midgut oocyst samples (**C**) and twofold serially diluted salivary gland sporozoite samples (**D**) by bCUBE. *R*^2^ represents the correlation coefficient of the slope, Ct the cycle threshold, and ns, not significant (*P* > 0.05)

The results indicated no significant difference in the levels of infection prevalence, as determined by the two different methods, in terms of both oocyst (Fig. 2A) and sporozoite detection (Fig. 2B). At both low and high infection intensities, the infection prevalence values as determined by the bCUBE- and microscopy-based methods were comparable, suggesting that bCUBE qPCR is as reliable as microscopy for detecting *P. falciparum*-infected mosquitoes. Specifically, in the low-infection intensity cohorts, oocyst prevalence was determined to be 79.8% by the bCUBE-based assays and 80.4% by microscopy; in the high-infection cohorts, the prevalence was 100% by the bCUBE assay and 98.5% by the microscopy-based assay. Similarly, sporozoite infection prevalence in the low-infection intensity group was determined to be 81.1% by the bCUBE-based assay and 69.4% by the microscopy-based assay, and in the high-infection intensity group, it was determined to be 92.9% by the bCUBE-based assay and 95.1% by the microscopy-based assay.

We further assessed the ability of the bCUBE system to detect low-level *P. falciparum* infections in microscopy-confirmed midgut DNA samples. The bCUBE qPCR assays successfully detected as few as one oocyst per midgut in these samples. However, regression analysis revealed a relatively lower correlation between Ct values and the number of oocysts per midgut (*R*^2^ = 0.856) (Fig. 2C), indicating a weaker linear relationship. In addition, we compared sporozoite loads determined by microscopy to those from bCUBE qPCR assays using serially diluted sporozoite DNA samples. The bCUBE qPCR showed strong sensitivity, detecting as few as 5–10 sporozoites per sample, with a Ct value of 37.0, which was below the established threshold of 39.0. A strong correlation was observed between Ct values and sporozoite numbers, with an *R*^2^ value of 0.968 (Fig. 2D), confirming the capacity of the bCUBE qPCR assays to detect and quantify sporozoite loads in mosquitoes and indicating its potential for use in field-based vector surveillance.

These results demonstrate that the bCUBE qPCR system is a reliable and sensitive tool for detecting *P. falciparum* infections in mosquitoes that is comparable to conventional microscopy, even at low infection intensities. Thus, it appears to be a viable option for field-based malaria transmission monitoring and vector surveillance.

### bCUBE qPCR-based detection of *P. falciparum* in mosquito pools

**Fig. 3:**
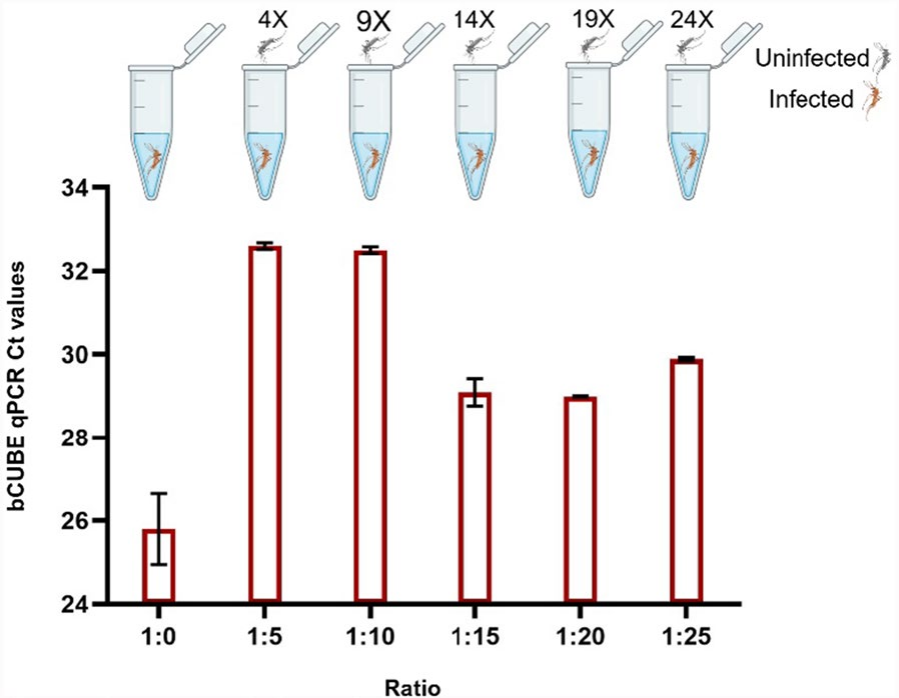
bCUBE-based detection of *P. falciparum* infection in pooled *An. gambiae* mosquitoes. Five different pool ratios were prepared, each containing a single *P. falciparum*-infected mosquito combined with various numbers of uninfected mosquitoes (ratios: 1:5, 1:10, 1:15, 1:20, and 1:25). A pool of 25 uninfected mosquitoes served as a negative control, while a single infected mosquito (1:0) was used as a positive control. The upper panel illustrates the experimental setup, showing the number of uninfected mosquitoes in each pool. The lower panel shows the bCUBE qPCR cycle threshold (Ct) values, indicating consistent detection of *P. falciparum* infection across all pooled ratios

We then evaluated the performance of the bCUBE-based real-time PCR system in detecting *P. falciparum* in pooled mosquito samples of various ratios. Five different pool sizes were prepared, each containing one *P. falciparum*-infected mosquito mixed with uninfected mosquitoes in ratios ranging from 1:5 to 1:25. A pool of 25 uninfected mosquitoes was used as a negative control, and a single infected mosquito served as a positive control. *P. falciparum* was successfully detected across all pooled ratios, with the positive control displaying a strong detection signal (Ct value: 26.0 ± 0.84). As expected, the Ct values increased as the number of uninfected mosquitoes in the pool increased, reflecting a decrease in the relative proportion of infected material. These findings suggest that the bCUBE qPCR assay is highly sensitive and capable of detecting *P. falciparum* in pooled samples (Fig. 3).

Interestingly, the highest Ct values were observed for the lower-ratio pools (1:5 and 1:10), with average Ct values of 32.6 and 32.5, respectively, as compared to the slightly lower Ct values observed for the higher-ratio pools (1:15, 1:20, and 1:25), which ranged between 29.0 and 29.9. This counterintuitive trend could be explained by several factors, including variations in infection intensities of the single-infected mosquitoes used in the lower-ratio pools and inconsistencies in the DNA extraction efficiency. Differences in parasite load within individual infected mosquitoes may lead to lower amounts of detectable DNA in some samples, causing a higher Ct value even in the lower ratios. Minor variations in DNA extraction procedure or sample processing could also contribute to these discrepancies, resulting in less DNA being captured from the infected mosquito in the smaller pools, thereby increasing the Ct values.

### bCUBE-qPCR detects mixed *Plasmodium* species infections

bCUBE qPCR assays were used to detect mixed *Plasmodium* species infections by means of a multiplex PCR approach with TaqMan probes. Two assays were performed: a duplex PCR for detecting *P. falciparum* and *P. vivax* using FAM and HEX fluorescent probes, respectively, and a triplex PCR for detecting *P. malariae*, *P. ovale*, and *P. knowlesi* using FAM, HEX, and CY5 fluorescent probes. The performance of these assays was evaluated using mixtures of plasmid DNA containing the 18S rRNA gene from each *Plasmodium* species at various concentrations.

In the duplex assay, *P. falciparum* and *P. vivax* were detected both individually and in mixed infections. *Plasmodium falciparum* showed consistent detection, with Ct values ranging from 19.2 ± 0.07 to 28.8 ± 0.07, depending on the plasmid concentration. Similarly, *P. vivax* was detected with Ct values ranging from 18.0 ± 0.17 to 27.9 ± 0.21. However, when the *P. falciparum* plasmid concentration was increased to 10^5^ or 10^6^ copies, *P. vivax* was no longer detectable, indicating that the detection limit for *P. vivax* was affected by the high concentration of *P. falciparum* in mixed infections. This suggests potential competition between the two targets at high *P. falciparum* concentrations, adversely affecting the detection of *P. vivax* (Table 2).

**Table 2:** Duplex bCUBE-qPCR-based detection of *P. falciparum* and *P. vivax* in mixed plasmid templates targeting *Plasmodium* 18S rRNA gene

*P. falciparum* (18S rRNA) copy number	*P. vivax* (18S rRNA) copy number	Duplex bCUBE qPCR cycle threshold ± standard deviation	
		*P. falciparum *(FAM)	*P. vivax *(HEX)
10^3^	0	28.8 ± 0.07	NTD
10^4^	0	25.7 ± 0.06	NTD
10^5^	0	22.2 ± 0.14	NTD
10^6^	0	19.2 ± 0.07	NTD
0	10^3^	NTD	27.9 ± 0.21
0	10^4^	NTD	24.4 ± 0.24
0	10^5^	NTD	21.1 ± 0.01
0	10^6^	NTD	18.0 ± 0.17
10^3^	10^3^	29.6 ± 0.14	28.7 ± 0.07
10^3^	10^4^	29.5 ± 0.07	25.8 ± 0.07
10^3^	10^5^	31.2 ± 0.99	22.3 ± 0.21
10^3^	10^6^	36.2 ± 0.57	18.7 + 0.21
10^4^	10^3^	26.3 ± 0.07	27.8 ± 0.29
10^5^	10^3^	22.7 ± 0.28	NTD
10^6^	10^3^	19.8 ± 0.14	NTD

*NTD* not detected

In the triplex assay, *P. malariae*, *P. ovale*, and *P. knowlesi* were detected across varying plasmid concentrations without cross-interference. *Plasmodium malariae* was consistently detected, with Ct values ranging from 18.4 ± 0.16 to 32.7 ± 1.23. However, the detection limit for *P. ovale* was dramatically affected when the plasmid concentration of *P. malariae* was increased to 10^6^ copies, at which point *P. ovale* became undetectable. Similarly, *P. knowlesi* detection was also affected at high concentrations of *P. malariae*, with *P. knowlesi* becoming undetectable when *P. malariae* was present at 10^5^ or 10^6^ copies. These results suggest that at higher concentrations of *P. malariae*, the detection of *P. ovale* and *P. knowlesi* is inhibited, likely because of competition between targets (Table 3).

**Table 3:** Triplex bCUBE-qPCR-based detection of *P. malariae, P. ovale,* and *P. knowlesi* in mixed plasmid templates targeting *Plasmodium* 18S rRNA gene

*P. malariae* (18S rRNA) copy number	*P. ovale* (18S rRNA) copy number	*P. knowlesi* 18S rRNA) copy number	Triplex bCUBE qPCR cycle threshold ± standard deviation		
			*P. malariae* (FAM)	*P. ovale* (HEX)	*P. knowlesi* (CY5)
10^3^	0	0	28.7 ± 0.04	NTD	NTD
10^4^	0	0	24.6 ± 0.17	NTD	NTD
10^5^	0	0	22.0 ± 0.05	NTD	NTD
10^6^	0	0	18.4 ± 0.16	NTD	NTD
0	10^3^	0	NTD	28.9 ± 0.57	NTD
0	10^4^	0	NTD	25.3 ± 0.11	NTD
0	10^5^	0	NTD	21.9 ± 0.04	NTD
0	10^6^	0	NTD	18.7 ± 0.18	NTD
0	0	10^3^	NTD	NTD	29.1 ± 0.25
0	0	10^4^	NTD	NTD	25.6 ± 0.15
0	0	10^5^	NTD	NTD	23.1 ± 0.68
0	0	10^6^	NTD	NTD	18.7 ± 0.12
10^3^	10^3^	10^3^	29.7 ± 0.30	29.7 ± 0.41	32.5 ± 0.67
10^3^	10^3^	10^4^	30.0 ± 0.13	30.5 ± 0.19	29.0 ± 0.24
10^3^	10^3^	10^5^	30.0 ± 0.17	30.0 ± 0.33	25.0 ± 0.15
10^3^	10^3^	10^6^	32.7 ± 1.23	32.0 ± 2.40	22.0 ± 1.23
10^3^	10^4^	10^3^	30.1 ± 0.20	27.0 ± 0.02	34.6 ± 1.06
10^3^	10^5^	10^3^	32.8 ± 0.81	23.7 ± 0.05	37.7 ± 0.51
10^3^	10^6^	10^4^	34.9 ± 0.27	20.2 ± 0.23	NTD
10^4^	10^3^	10^3^	25.7 ± 0.50	29.8 ± 0.50	37.4 ± 0.50
10^5^	10^3^	10^3^	23.6 ± 0.08	30.3 ± 2.81	NTD
10^6^	10^3^	10^3^	19.5 ± 0.09	NTD	NTD

*NTD* not detected

These findings highlight the bCUBE qPCR assay’s ability to detect mixed *Plasmodium* species infections using a TaqMan probe-based multiplex PCR. However, high concentrations of one species, such as *P. falciparum* in the duplex assay or *P. malariae* in the triplex assay, may limit the detection of other species (*P. vivax*, *P. ovale*, or *P. knowlesi*) in mixed-species infections. This potential competition between targets should be considered in diagnostic and field surveillance applications.

### bCUBE qPCR can detect *P. falciparum* infection of mosquitoes under field conditions

To evaluate the feasibility of using the bCUBE qPCR system for malaria parasite surveillance in the field, we conducted a pilot field study involving malaria-endemic regions of Cameroon. All experimental assays, including mosquito collection, morphological identification, DNA isolation, and bCUBE qPCR using 18S rRNA primers and probes, were completed at the field sites within 4–5 h from the initiation of mosquito collection. This rapid workflow highlights the practicality of deploying the portable bCUBE system for field diagnostics.

**Fig. 4:**
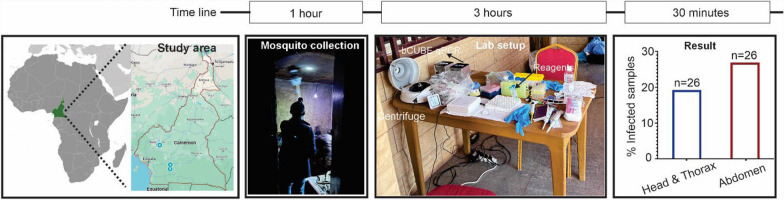
Pilot field testing of *P. falciparum* infection assays in field-collected *Anopheles* mosquitoes. The feasibility of the bCUBE machine for human malaria parasite surveillance was assessed in a preliminary field study in the Cameroon malaria-endemic region. Mosquito collection, sorting of mosquitoes, DNA isolation, and bCUBE qPCR using 18S rRNA primers/probe were all performed at the field site within 4–5 h. The graph indicates that 23% of abdomen and 19% of head + thorax samples of collected mosquitoes were positive for *P. falciparum* infection

We tested two DNA isolation methods, DNAzol and Squash buffer, for field-based DNA extraction. While both methods were functional in the field, the DNAzol proved more stable at room temperature, whereas the Squash method required proteinase K, which needs to be stored at 4 °C. The bCUBE-based diagnostic system successfully detected *P. falciparum* infection in both the abdomen and head + thorax samples from the collected mosquitoes, demonstrating its utility for in-field malaria surveillance. The infection prevalence detected using bCUBE qPCR assays was 19.2% for head + thorax samples and 26.9% for abdomen samples (Fig. 4). In contrast, conventional qPCR analysis in the main laboratory showed an infection prevalence of 23.1% in abdomen samples but failed to detect *P. falciparum* in any head + thorax samples.

These findings suggest that the portable bCUBE qPCR platform offers comparable sensitivity to conventional benchtop qPCR assays in detecting *P. falciparum* infections in mosquito abdomens but may outperform conventional qPCR platforms in detecting infections in head + thorax samples. The presence of *P. falciparum* in head + thorax samples indicates potentially infectious mosquitoes, underscoring the value of the bCUBE-based diagnostic system for rapid field-based detection and transmission monitoring.

## Discussion

Malaria diagnosis and surveillance are traditionally carried out using microscopy and standard laboratory benchtop qPCR equipment. These approaches are highly sensitive but impractical for field use because of their need for laboratories and highly trained personnel. To overcome these limitations, the use of portable qPCR systems, such as the bCUBE, has been explored as a potential field-deployable solution. Building on earlier work that validated the bCUBE qPCR for arbovirus and *Wolbachia* detection in *Aedes aegypti* mosquitoes [13], we have now demonstrated its feasibility for detecting *P. falciparum* using in vitro-cultured parasites in human blood and experimentally infected mosquitoes, as well as for field-collected mosquito samples.

For the detection of other *Plasmodium* species (*P. vivax, P. ovale, P. malariae,* and *P. knowlesi*), we used plasmid DNA containing the species-specific 18S rRNA gene as a proxy, given the challenges associated with in vitro culturing of these species and obtaining patient infected blood samples, unlike *P. falciparum*, which can be maintained in continuous culture [25]. While plasmid-based validation is a widely accepted alternative [26], we acknowledge that future studies incorporating clinical samples from malaria-endemic regions will serve as a more realistic reference material to standardize the performance of the diagnostic method for such species.

Validation of the bCUBE-based qPCR assays against those performed with the established laboratory-standard StepOnePlus qPCR machine showed high concordance between the two platforms, with a strong linear correlation (*R*^2^ = 0.993), indicating that the bCUBE-based assays can achieve comparable sensitivity. Moreover, the bCUBE-based assays were able to detect *P. falciparum* DNA at concentrations as low as 0.5 parasites/µl, which is critical for early-stage malaria detection. This sensitivity aligns with previous studies that have established qPCR as a more sensitive method than microscopy for detecting low parasitemia, which is often missed with traditional diagnostic tools [27]. In addition to their sensitivity, the bCUBE qPCR assays demonstrated high reliability for detecting *P. falciparum* infections in mosquito samples when compared to microscopy. Our results showed no significant difference in determined infection prevalence between the two methods for detecting oocysts and sporozoites, indicating that the bCUBE-based assays can be used as an alternative to microscopy for vector infection surveillance. This finding is consistent with a report by Rutkowski et al. [13] that showed that bCUBE-based assays can be effectively used for arbovirus detection in *Aedes* mosquitoes. The detection of low-level infections, such as a single oocyst or sporozoite, is crucial for understanding transmission dynamics in vector populations [28, 29]. This diagnostic platform was also able to detect a single *P. falciparum*-infected mosquito when pooled with 4, 9, 14, 19, or 24 uninfected mosquitoes in three biological replicates, in line with findings from other pooling strategies that have been used to increase the efficiency of vector surveillance programs [11]. This capacity underscores the potential of the bCUBE-qPCR for large-scale surveillance of malaria vectors when pooling is necessary to manage high sample volumes at low cost.

The field-deployable nature of the bCUBE qPCR assays was further validated through a pilot study conducted in malaria-endemic regions of Cameroon. We successfully detected *P. falciparum* infections in both the abdomen and head + thorax of collected mosquitoes on-site, demonstrating that the bCUBE qPCR assays can generate same-day results, thereby reducing the time and cost associated with transporting samples to central laboratories. During the field study, mosquitoes were collected and processed on-site, in a hotel room, where the sample preparation and bCUBE qPCR assay were performed. Due to logistical constraints, microscopy was not performed for parasite detection. Instead, the field-tested samples were later analyzed using a benchtop qPCR in a central laboratory, which yielded comparable results for abdomen samples, indicating consistent detection in that compartment. In contrast, for head + thorax samples, the bCUBE system consistently detected infections that were missed by conventional qPCR. These discrepancies are likely due to differences in platform sensitivity and potential DNA degradation during storage and transport to the central laboratory. Notably, the bCUBE system demonstrated high sensitivity by detecting as few as 5–10 sporozoites, a capability that is critical for timely interventions in malaria control programs, especially in remote areas where conventional laboratories are lacking.

### A low-cost qPCR assay for field-based malaria parasite detection

The primary goal of this study was to develop and optimize a cost-effective qPCR assay for detecting human malaria parasites in both human and mosquito samples using the portable bCUBE-qPCR platform. While microscopy remains the gold-standard and cost-effective method for malaria diagnosis, it is labor-intensive and requires highly skilled personnel, making it less practical for large-scale field surveillance. A major concern for field-based surveillance programs is the high initial cost of qPCR machines and the logistical challenges associated with transporting samples to a central laboratory. The bCUBE is 50–80% more affordable than standard benchtop qPCR systems like the StepOnePlus and the QuantStudio Pro. In addition, the bCUBE device’s low power consumption (20 W average, 60 W maximum) allows it to operate via a small 1.5-kg battery pack with a 4–5 h runtime, making it ideal for remote field use, where the power supply may be limited. This setup significantly reduces the operational burden when compared with conventional qPCR machines that typically require 850 W.

A common practice in field-based malaria studies is to collect samples and transport them to a central laboratory for analysis. This process involves extensive logistics, including storing the samples under cold conditions during transport to a central laboratory, incurring high costs and possibly delaying results for 2–4 months. In contrast, using the portable bCUBE qPCR enables the generation of results on the same day a sample is collected. This rapid turnaround time is a key advantage for real-time malaria surveillance and decision-making in endemic areas. In addition, the bCUBE’s cloud-based data platform allows results to be accessed remotely, making it possible for researchers and health officials to monitor data from anywhere.

The total time required for sample collection, DNA extraction, bCUBE-qPCR assay, and data analysis is approximately 4–5 h. To further improve field applicability, pre-loaded reagent cartridges are being developed that can be stored at ambient temperature for up to 1 year for *Plasmodium* species detection. These cartridges will contain reaction master mix along with primer and probes. This advancement would eliminate the need for cold storage, allowing users to simply add the DNA sample and buffer directly to the dry cartridge to initiate the test, thereby simplifying the workflow and reducing costs.

While the current bCUBE qPCR system has notable advantages, it also has some limitations. The 36-well cartridge restricts the number of samples that can be processed in a single run, making it less efficient for large-scale studies than the 96-well plates used in conventional benchtop qPCR devices. However, the ability to perform multiplex assays using hydrolysis probes for simultaneous detection of multiple *Plasmodium* species offsets this lower throughput.

Overall, the bCUBE system offers a practical, rapid, and field-deployable solution for malaria parasite detection, providing same-day results and cloud-based accessibility, making it an ideal tool for real-time surveillance in remote and low-resource settings.

## Conclusions

We have developed a portable, cost-effective, and sensitive qPCR-based diagnostic platform using the Hyris bCUBE system for detecting *Plasmodium* species. This system accurately detects five *Plasmodium* species, including *P. falciparum*, even at low parasite densities, and shows a strong correlation with standard laboratory qPCR. Its portability, low cost, and easy-to-use DNA extraction method make it ideal for field deployment in resource-limited settings. The ability to provide same-day, on-site results reduces the need for centralized laboratory testing, supporting timely malaria surveillance and control decisions. Additionally, its compatibility with pooled mosquito surveillance offers a cost-effective solution for large-scale vector monitoring. Overall, the bCUBE qPCR system is a promising tool for malaria diagnostics and mosquito-borne disease surveillance, especially in remote and low-resource areas.

## Data Availability

No datasets were generated or analyzed during the current study.

## Abbreviations

qPCR: Quantitative polymerase chain reaction
BEI Resources: Biodefense and Emerging Infections Research Resources Repository
RDTs: Rapid diagnostic tests
HRP: Histidine-rich protein
LAMP: Loop-mediated isothermal amplification
rRNA: Ribosomal ribonucleic acid
